# The influence of anxiety and fear of COVID-19 on vaccination hesitancy among postsecondary students

**DOI:** 10.1038/s41598-022-25221-2

**Published:** 2022-11-29

**Authors:** Andrej Šorgo, Nuša Crnkovič, Katarina Cesar, Špela Selak, Mitja Vrdelja, Branko Gabrovec

**Affiliations:** 1 Faculty of Natural Science and Mathematics, University of Maribor, Koroška Cesta 160, 2000 Maribor, Slovenia; 2 National Institute of Public Health, Trubarjeva 2, 1000 Ljubljana, Slovenia

**Keywords:** Diseases, Health care, Health occupations, Medical research, Risk factors, Signs and symptoms

## Abstract

The aim of the present study was to explore the influence of anxiety and fear of COVID-19 on vaccination hesitancy among Slovenian postsecondary students. A cross-sectional study using a set of previously tested instruments and ad hoc questions created by the authors was chosen as the method to gain insight into various health and sociodemographic aspects of Slovenian postsecondary students affected by the COVID-19-induced closures and suspensions of educational activities at tertiary educational institutions (N = 5999). Overall, 39.7% of participating students expressed an intention to get vaccinated at the first possible opportunity, whereas 29.2% expressed no intent to do so. The highest vaccine hesitancy was observed among prospective teachers (50.3%) and the lowest among prospective physicians (5,7%). When examining the role of anxiety and fear of COVID-19 on the Slovenian postsecondary students’ intentions to get vaccinated the results of logistic regression showed that only fear of COVID-19 played a mild and significant role.

## Introduction

Vaccination and vaccination campaigns are widely recognized as the greatest public health achievements in human history^1,2^. Yet, despite solid evidence that the benefits of vaccination far outweigh any potential harms, there is a worldwide hesitancy to vaccinate^3–5^ resulting in unnecessary health problems and morbidity from preventable diseases. The problem is so severe that vaccine hesitancy has been named one of the top ten global health problems by the World Health Organisation^6^. In addition to existing surveys on vaccines, such as the measles vaccine, vaccine hesitancy and even resistance to vaccines and vaccination was again demonstrated in the COVID-19 pandemic caused by severe acute respiratory syndrome coronavirus 2 (SARS-CoV-2)^7^.

According to the official data, the first infection with a novel coronavirus was registered in Slovenia on the 4th of March 2020 and the vaccination campaign started on the 27th of December 2020 for the vaccination of people older than 80 years, health care workers and residents of nursing homes. At the time of questionnaire submission (February–March 2021), the first doses of vaccine were available to the most vulnerable population groups aged from 18 to 65 years, which included vulnerable postsecondary students as a target population. However, the decision to get vaccinated was a voluntary decision of each citizen in Slovenia^8–10^. While there are parts of the world where vaccines against COVID-19 are still not fully available, there are also parts of the world, especially in rich countries like Slovenia, where people can choose to get vaccinated and the availability of vaccines is not a reason not to get vaccinated^11^. This raises the questions: why do so many people refuse vaccination, and secondly, what are the reasons for the refusal.

As postsecondary students are of legal age, they are fully responsible for their own decisions, including the decision to get vaccinated or not. Research shows that the prevalence of vaccination hesitancy among postsecondary students ranges between 13 and 19.3%^12,13^. Lower levels of vaccination hesitancy were observed among medical students—10.6% in India^14^. On the other hand, the global prevalence of COVID-19 vaccination hesitancy among students/trainees in healthcare were reported to be 18.9%^15^. Even higher levels of COVID-19 vaccination rejection was found among dental students in a global study involving 22 countries—22.5% were hesitant and 13.9% rejected the vaccine^16^. However, the refusal to be vaccinated raises additional questions as higher levels of fear relating to the infection with SARS-CoV-2 are reported among postsecondary students compared to other age groups^17^. Moreover, a positive correlation between fear of COVID-19 and anxiety was also observed, especially among female students^17,18^. Thus, an argument can be made that given their higher levels of fear of COVID-19 and anxiety they would be more inclined to get vaccinated for COVID-19, which was also found by various studies^19–21^.

Yet many factors can negatively influence the intentions and implementation of vaccination campaigns, which can have devastating effects not only on the health of individuals but also on society as a whole. Therefore, an understanding of the factors that contribute to vaccination hesitancy is essential. The majority of postsecondary students’ physical health may not be as affected by COVID-19 as more vulnerable groups are (e.g., elderly, immunocompromised, chronically ill), but they are an active part of the community and potentially active spreaders of the virus. Moreover, they will act as gatekeepers or promoters of maladaptive behaviours in the future, as many of them will act as influencers and decision makers. This applies, for example, to the use of other vaccines against life-threatening diseases for their children or future behaviours in response to new outbreaks.

Therefore, the aim of the present study is to examine the influence of fear of COVID-19 and anxiety on Slovenian postsecondary students’ intentions to get vaccinated when the vaccine would be available. To the best of our knowledge is the first study that examined the entire post-secondary student population in Slovenia.

The results may serve as a basis for a better understanding of vaccination hesitancy among students and enable the development of targeted interventions and campaigns against it. The research questions that guided our study were as follows:

RQ 1: How great are anxiety and fear among the students?
RQ2: Are students a one-dimensional population in terms of intent to get vaccinated against COVID-19?
RQ 3: Can anxiety and fear of COVID-19 be used as predictors of willingness to get vaccinated or not?
RQ 4: Is there a difference between medicine and healthcare students in terms of their intentions relating to vaccination against COVID-19?

## Methodology

### Participants and procedure

The present study is part of the research titled “Students’ experience of COVID-19 epidemic” aimed to assess and understand the impact of COVID-19 and related measures on the postsecondary students’ mental health^22,23^. A cross-sectional study was designed comprising psychological instruments, sociodemographic questions and ad hoc questions created by authors. All methods were carried out in accordance with relevant guidelines and regulations.

The participants were postsecondary students enrolled in Slovenian’ higher education institutions. Thus, for the present study participants that were at the time of the study enrolled as full-time students were assessed as eligible. At the time of the present study that included approximately 60.600 students^24^. They were recruited online via a web-based survey platform (https://www.1ka.si/); the data collection took place between the 9th of February and the 8th of March 8 2021, on the whole territory of Slovenia. Simple random sampling was used—invitation letters to participate in the study were sent to all universities, private faculties, and student organisations with a request to forward the invitation to all their students. To get as much feedback as possible a reminder letter with the invitation to participate was sent to all addressees after one week and after another week to those from whom we had not received any feedback.

At the beginning of the questionnaire, the participants were informed about various aspects of the study, including their rights to voluntarily participate or withdraw from the study and that the data will be processed in accordance with EU and Slovenian legislation. Thereafter the participants were asked to give consent to participate in the research and state that they have read the information regarding the research they intend to participate in. Therefore, informed consent was obtained from all the participants before they filled out the questionnaire in the present study. The sample structure is presented in Table 1.

**Table 1:** Demographic properties of the sample (N = 5999).

Demographic characteristics	Frequency (%)
**Gender**	
Female	4347 (72.5%)
Male	1598 (26.2%)
Other	53 (0.9%)
Missing	1
**Educational level**	
Higher vocational	190 (3.2%)
Bachelor	3694 (61.6%)
Master	2083 (34.7)
Doctoral	23 (0.4%)
Other	8 (0.1%)
Missing	1

Ethical approval to conduct the study was obtained from the National Medical Ethics Committee of the Republic of Slovenia (NMEC), Ministry of Health (No. 0120-48/2021/3). The entire methodology—from creating the questionnaire to delivery and processing of the data was conducted in accordance with ethical and professional guidelines and regulations.

### Instruments and questions

The Fear of COVID-19 Scale (FCV-19S)^25^ is a seven-item scale assessing fear of COVID-19 (hereafter: FCOV). The seven items (see Table 4) are scored on a 5-point Likert scale ranging from 1 (strongly disagree) to 5 (strongly agree), with scores ranging from 7 to 35. The higher the score, the greater the fear of COVID-19. According to references^25,26^ the scale is unidimensional with strong psychometric characteristics and reliability expressed by Cronbach’s alpha above 0.80 in the general population as well in the population of postsecondary students in the interest of the study^27,28^. In some studies, a different factorial structure was found. For example, Reznik and colleagues^28^ reported two components based on Principal Component Analysis with Varimax rotation.

The Generalized Anxiety Disorder Questionnaire (GAD-7)^29^ is a 7-item self-report measure to assess the severity of anxiety and its symptoms according to DSM-IV criteria. Participants rated how often they experienced anxiety symptoms in the past 2 weeks on a 4-point Likert scale from 0 (not at all) to 3 (almost every day). Total scores range from 0 to 21, with a cut-off score of 10 identifying instances of generalized anxiety disorder. The following cut-offs correlate with the level of anxiety severity and scores ≥ 5, ≥ 10, and ≥ 15 are representing mild, moderate and severe anxiety symptom levels.

Intention to be vaccinated against COVID-19 was examined by asking the participants “Do you intend to get vaccinated?”. Three responses were offered, and they should tick one of them:

Yes, I will vaccinate at the first opportunity.

Yes, I will vaccinate, but later.

No, I do not intend to be vaccinated.

The responses were scored 2, 1, and 0, respectively. At the time of the data collection (February–March 2021) and in line with the vaccination plans communicated by the Slovenian authorities, postsecondary level students were not being vaccinated. The exception to the rule was a minority of students with a chronic disease with the risk of severe complications caused by the infection, for all the others vaccination was only foreseen in some uncertain future. Therefore, it was not possible to measure their actual behaviour (to opt, opt-out or postpone decision) based on the actual offer of vaccination but their behavioural intention^30^ toward something that was an option to happen in an uncertain future.

Sociodemographic data examined in the present study included gender, field of study and educational level.

### Statistical analyses

Constructs anxiety (ANX) measured by GAD-7 and fear of Covid-19 (FCOV) assessed by FCV-19S, were handled in a similar way. Because the sums of items in the constructs were of interest after an initial data screening, data sets of respondents with missing data were listwise deleted from the poll. Each variable was screened for measures of central tendencies, skewness, and kurtosis. Cronbach’s alphas were calculated as a measure of reliability. Before proceeding to Exploratory Factorial Analysis (EFA) the data matrices were inspected by use of KMO and Barlett’s test. With the application of Principal Component Analysis (PCA) with Direct Oblimin Rotation component (factor) structure of the constructs was explored.

A complete case analysis approach was applied, which resulted in a difference in the reported number of respondents in different analyses^31^. Cases with missing data were excluded from the poll by use of listwise deletion in correlational, regression, and exploratory factor analyses. Effect sizes were calculated as Eta square (η^2^) with cut-off values of 0.01 for small, 0.06 for medium, and 0.14 for large effect.

To the best of our knowledge, the FCV-19S was used in Slovenia for the first time and therefore, the initial validation was conducted. The choice was Principal Component Analysis (PCA) with a Direct Oblimin Rotation. The cut-off value of the Kaiser–Meyer–Olkin (KMO) measure of ≥ 0.8 was taken as empirical evidence of the suitability of the data matrix. Because items were conceptual similar even in the case of multiple factors, it was assumed they would be correlated^27^. For the FCV-19S assessment, Cronbach's alpha of 0.80, minimum inter-item correlations ranging from 0.15 to 0.50, and minimum corrected item-total correlations of 0.30 were used as indicators of internal consistency reliability.

Because we were not so much interested in the predictive role of each of the items forming scales the sum of items was used as a predictor of intention to be vaccinated. Binary logistic regression was a choice and the Spearman correlation coefficient was used to assess the connections between variables.

IBM SPSS® Version 28 was used to perform calculations. Additionally, we believed an EFA was more appropriate than a confirmatory factor analysis (CFA) because previous studies did not all find the same, one-factor solution across samples from different nations and languages. Furthermore, this is the recommended approach when conducting the first psychometric evaluation of a newly translated measure, regardless of the rigour of the translation method^32^. Given the conceptual similarity among items, we assumed that if there were multiple factors, they would be correlated. Thus, we conducted a PCA with a Direct Oblimin rotation. Based on recommendations by Clark and Watson^33^, we considered our sample size sufficiently large to conduct an PCA on the seven-item Fear of COVID-19 Scale. As recommended by Field^34^, we used a cut-off of the Kaiser–Meyer–Olkin (KMO) measure of ≥ 0.8 as empirical evidence of a sufficiently large sample size for factor analysis.

## Results

In Table 2, the results of postsecondary students’ intentions to be vaccinated are presented.

**Table 2:** Intentions of postsecondary students to be vaccinated.

Statement	Frequency	%	Valid %	Cumulative %
Yes, I will vaccinate at the first opportunity	2296	38.3	39.7	39.7
Yes, I will vaccinate later	1794	29.9	31.0	70.8
No, I do not intend to get vaccinated	1690	28.2	29.2	100.0
Total	5780	96.3	100.0	
Missing	219	3.7		
Total	5999	100.0		

Results are by no means encouraging because only approximately 40% of participants expressed intentions to be vaccinated at the first opportunity and about 30% reported not having any such intentions.

At the first glance at Table 3, it can be recognized that the highest proportion of students that have expressed the intention to be vaccinated are medical students. The result could be interpreted in the light of better health information and attitudes; however, the gap in the intentions to get vaccinated at the first opportunity between the medical students (82.9%) and students of healthcare (34.7%) is not easily commented in terms of misinformation. The highest number of students that expressed no intention to get vaccinated (50.3%) are prospective teachers.

**Table 3:** Differences between the study streams (N = 5779).

	n	Yes, first opportunity %	Yes, later %	No intentions %
Medicine	510	82.9	11.4	5.7
Science	1036	44.8	33.1	22.1
Humanistic	878	41.3	32.2	26.4
Art	512	37.3	30.5	32..2
Health care	629	34.7	31.3	34.0
Other	268	33.2	29.1	37.7
Social sciences (educational)	136	31.6	30.9	37.5
Social sciences (not educational)	1013	29.2	35.8	34.9
Technology and engineering	480	28.5	38.3	33.1
Criminal justice and security	164	22.6	29.3	48.2
Educational	153	22.2	27.5	50.3

The results are ordered by decreasing percentages of those who showed an intention to be vaccinated on the first opportunity.

*Note* Yes, I will vaccinate at the first opportunity. Yes, I will vaccinate, but later. No, I do not intend to be vaccinated.

### Validation of the Slovenian version of the Fear of COVID scale

In the analysis were included data provided by 4691 students for whom we collected all data of interest. Even, if possible, we do not apply data imputation but use listwise deletion instead. Cronbach’s alpha of the scale was 0.83, and deletion of any item will result in a lower value.

The value of Kaiser–Meyer–Olkin Measure of Sampling Adequacy (KMO) was 0.84 and the results of Bartlett's Test of Sphericity were: Chi-square = 13,425.03; *df* = 21; *p* < 0.001, which allows the intended factor analysis.

Even if it is supposed that the scale is unidimensional, the two components were extracted explaining about 58% of the variance (Table 4). Both components correlate (*r* = 0.561; *p* < 0.001). In the first component items are grouped to reflect physical harm or even death, where students generally disagree with such fears. In the second component items reflect psychological conditions, where numbers are much higher.

**Table 4:** Measures of central tendencies and results of Principal Component Analysis of the Slovenian version of the Fear of COVID-19 scale.

Code	Text	N	Mean	Median	Mode	SD	Skewness	Kurtosis	Comm	F1	F2
FCOVb	It makes me uncomfortable to think about coronavirus-19	4707	2.88	3.00	4	1.29	− 0.090	− 1.218	0.869		0.993
FCOVe	When watching news and stories about coronavirus-19 on social media, I become nervous or anxious	4709	2.70	3.00	1	1.33	0.138	− 1.227	0.401		0.572
FCOVa	I am most afraid of coronavirus-19	4709	2.33	2.00	2	1.10	0.442	− 0.613	0.486		0.461
FCOVc	My hands become clammy when I think about coronavirus-19	4706	1.46	1.00	1	0.79	1.912	3.688	0.458	0.582	
FCOVd	I am afraid of losing my life because of coronavirus-19	4705	1.43	1.00	1	0.79	2.134	4.699	0.493	0.672	
FCOVf	I cannot sleep because I’m worrying about getting coronavirus-19	4710	1.28	1.00	1	0.64	2.773	8.619	0.664	0.866	
FCOV1g	My heart races or palpitates when I think about getting coronavirus-19	4710	1.41	1.00	1	0.79	2.279	5.301	0.693	0.842	
Alpha	Cronbach's alpha									0.831	0.764
	Eigenvalue									3.703	1.091
	% Variance									47.068	10.985

Items are ordered by decreasing mean.

*SD* standard deviation, *Comm.* Communalities. *F1* Component loadings of the first principal component. *F1* Component loadings of the second principal component.

### Validation of the Slovenian version of the Generalized Anxiety Disorder questionnaire (GAD-7) scale

The value of Kaiser–Meyer–Olkin Measure of Sampling Adequacy (KMO) was 0.94 and the results of Bartlett's Test of Sphericity were as follows: Chi-square = 32,412.33; *df* = 21; *p* < 0.001, which allows intended factor analysis. Cronbach’s alpha of the GAD-7 within the present study is 0.940. The PCA was conducted and according to the results GAD-7 is a unidimensional tool, which confirms the findings of their authors, and the first component (Eigenvalue = 5.176) explains 73.94% of the variance. Due to the proprietary restrictions, analysis of individual items is not provided.

### Correlation between constructs ANX and FCOV

Spearman’s correlation coefficient rho between GAD-7 and FCV-19S (N = 4665) was 0.301 (CI95% 0.274–0.328) at the *p* < 0.001 level. Additionally, correlations between GAD-7 and both components emerging from FCV-19S, as a result of PCA (see Table 4) were calculated. It appears that sums calculated from items of both components (F1 = sum of FCOVcdeg; F 2 = sum of FCOVabe) correlate positively with GAD-7 (Rho = 0.301 (CI 0.273–0.327). More specifically, correlation between F2 and GAD-7 was a little bit higher (Rho = 0.301 (CI 0.273–0.327) than between F1 and GAD-7 (Rho = 0.235 (CI 0.206–0.262). All correlations were statistically significant at the *p* < 0.001 levels.

### Results of the logistic regression

The answer to the question about the intention to vaccinate (still postponed in the uncertain future at the time of the survey) was exclusive for each respondent. Consequently, the choice of one option excludes the other two options, so the binary logistic regression was chosen. Based on previous correlation analysis results, the independent variables in the equations were the sums of GAD-7 (ANX) and FCV-19S (FCOV) and each of the three options offered as outcome variables.

From the results of regression analyses (Betas), presented in Table 5, it can be concluded that Anxiety (ANX) and Fear of Covid-19 (FCOV) are most probably not major causes of vaccination hesitancy. Differences between the three groups are minor and statistically significant at *p* < 0.001 levels only for FCOV as positive predictor of intention to be vaccinated immediately, and negative in intentions not to get a vaccine. ANX is very weak predictor (*p* < 0.01) of rejection of vaccination.

**Table 5:** Results of binary logistic regression for the whole sample of tertiary students (N = 5779).

	B	S.E	Sig	Exp(B)
**Yes, I will vaccinate at the first opportunity**				
ANX	− 0.004	0.005	0.414	0.996
FCOV	0.051	0.007	< 0.001	1.053
Constant	− 1.020	0.106	< 0.001	0.351
**Yes, I will vaccinate later**				
ANX	− 0.008	0.005	0.095	0.992
FCOV	0.012	0.007	0.075	1.012
Constant	− 0.786	0.110	< 0.001	0.430
**No, I do not intend to get vaccinated**				
ANX	0.014	0.005	0.010	1.014
FCOV	− 0.081	0.008	< 0.001	0.922
Constant	− 0.117	0.118	0.831	0.978

*ANX* sums of GAD-7 scale, *FCOV* sums of FCV-19S scale, *B* (Beta) coefficient for the constant. *S. E.* standard error, *Sig.* significance, *Exp(B)* the odds ratio.

It can be revealed that differences in anxiety (*F* (N = 5392, *df* = 2) = 2.092, *p* = 0.123) are minor. Differences in FCOV scale (F (N = 4684, *df* = 2) = 60.343, *p* < 0.001) shows a decreasing trend, what can lead to a conclusion that fear is a mild driver toward vaccination. The finding is supported by the value of Eta squared (0.025, CI 0.017–0.034), which indicates a small effect.

We continued with the analysis of data from two subgroups, namely prospective physicians (medical students) and prospective healthcare workers, which in our opinion will be most influential on the general public’s intentions to be vaccinated.

Upon examining the results of logistic regression (Table 6), it can be observed that ANX and FCOV have a statistically significant (*p* < 0.05) influence on their decision to get vaccinated as soon as possible but not them who decided to postpone it. Additionally, FCOV is a significant (*p* < 0.05) negative predictor for reporting no intentions to get vaccinated.

**Table 6:** Results of binary logistic regression for the sample of medical students (prospective physicians) (N = 510).

	B	S.E	Sig	Exp(B)
**Yes, I will vaccinate at the first opportunity**				
ANX	− 0.049	0.022	0.023	0.952
FCOV	0.111	0.038	0.003	1.118
Constant	0.691	0.479	0.149	2.002
**Yes, I will vaccinate later**				
ANX	0.036	0.026	0.175	1.037
FCOV	− 0.061	0.044	0.162	0.941
Constant	− 1.748	0.574	0.002	0.173
**No, I do not intend to get vaccinated**				
ANX	0.056	0.032	0.081	1.058
FCOV	− 0.167	0.061	0.006	0.845
Constant	− 1.187	0.741	0.109	0.305

*ANX* sums of GAD-7 scale, *FCOV* sums of FCV-19S scale, *B* coefficient for the constant. *S. E.* standard error, *Sig.* significance, *Exp(B)* the odds ratio.

Similar to the results of the entire test population, there were small and statistically not significant differences in terms of levels of ANX (Eta squared: 0.004, CI 0.00–0.020; *p* = 0.404) and FCOV (Eta squared: 0.018, CI 0.00–0.048; *p* = 0.029) among medical students as well (Table 6).

As evident in Table 7, FCOV had a statistically significant effect at p < 0.001 on healthcare students’ intent not to get vaccinated, but not on their intentions to obtain the vaccination. ANX once again did not play a statistically significant (p > 0.001) role in students’ expressed intentions to get vaccinated or to decline the vaccination.

**Table 7:** Results of binary logistic regression for the sample of prospective healthcare workers (N = 629).

	B	S.E	Sig	Exp(B)
**Yes, I will vaccinate at the first opportunity**				
ANX	− 0.024	0.015	0.118	0.974
FCOV	0.066	0.020	0.001	1.070
Constant	− 1.265	0.277	< 0.001	0.286
**Yes, I will vaccinate later**				
ANX	− 0.001	0.015	0.923	1.000
FCOV	0.022	0.020	0.281	1.023
Constant	− 0.979	0.277	< 0.001	0.365
**No, I do not intend to get vaccinated**				
ANX	0.027	0.016	0.086	1.028
FCOV	− 0.103	0.023	< 0.001	0.898
Constant	0.299	0.298	0.317	1.394

*ANX* sums of GAD-7 scale, *FCOV* sums of FCV-19S scale, *B* coefficient for the constant, *S. E.* standard error, *Sig.* significance, *Exp(B)* the odds ratio.

By examining the results of ANOVA test, it can be observed in Table 8 that differences in anxiety levels (*F* (N = 5392, df = 2) = 0.234, *p* = 0.791) were small (Eta squared: 0.001, CI 0.00–0.008, *p* = 0.404). However, the differences in FCOV scale (*F* (N = 5392, df = 2) = 9.501) were statistically significant (*p* < 0.001) with a small to medium effect. The finding is supported by the value of Eta squared (0.036, CI 0.009–0.070).

**Table 8:** Results of ANOVA test between three groups according the intention to get vaccinated.

Sum of Squares	df	Mean Square	F	Sig
**ANX1sum0**				
Between groups	20.127	2	10.064	0.791
Within groups	25,127.238	585	42.953	
Total	25,147.366	587		
**FCOVsum**				
Between groups	449.209	2	9.501	< 0.001
Within groups	12,127.510	513	23.640	
Total	12,576.719	515		

*ANX* sums of GAD-7 scale, *FCOV* sums of FCV-19S scale, *df* degree of freedom, *F* F distribution value. *Sig.* significance.

## Discussion

The aim of the present study was to explore the influence of anxiety and fear of COVID-19 on vaccination hesitancy among Slovenian postsecondary students. The data collection used in the present study was conducted in February 2021 when the vaccine was not fully accessible to the general population, but was distributed in line with the governmental priority plan. Thus, in the present study, the postsecondary students’ intentions for future behaviour were measured. More specifically, their intentions to get vaccinated at the first opportunity, to get vaccinated at a later point or not get vaccinated.

Overall, 39.7% of participating students expressed an intention to get vaccinated at the first possible opportunity, whereas 29.2% expressed no intent to do so. These results are not encouraging as the level of vaccine hesitancy among postsecondary students reported by other researchers rage between 13% and 19.3%^12,13^ Although in the present study the highest vaccine hesitancy was observed among prospective teachers (50.3%), the levels of vaccine hesitancy among participating healthcare students were almost twice as high compared to the results of the review that involved studies from 39 countries—34% vs. 18.9% respectively^15^. On the other hand, only 5.7% of participating medical students (which involved also dental medicine students) reported no intentions to get vaccinated. This percentage was much lower compared to the Indian medical students where 10.6% expressed vaccine hesitancy and dental students where 13.9% rejected the vaccine and 22.5% expressed hesitancy^16^.

When examining the role of anxiety and fear of COVID-19 on the Slovenian postsecondary students’ intentions to get vaccinated the results showed that only fear of COVID-19 played a mild and significant role. More specifically, when students reported higher levels of fear of COVID-19 they expressed less vaccine hesitancy intentions. Similar results were observed upon inspection of intentions to get vaccinated among healthcare students and medical students.

Moreover, anxiety was found to have a significant role in expressed intentions to get vaccinated at the first possible opportunity only among the medical students, and played a weak statistically significant role among tertiary students’ group that reported no intentions to obtain the vaccination when it will become accessible. However, these weak statistically significant effects could also have emerged due to the large sample size, which could potentially cause small differences to become statistically significant. Indeed other researchers also reported fear of COVID-19 to have a role in the vaccination hesitancy among postsecondary students, but they have found anxiety to have a significant role as well^17–19,21^.The difference in the reported influence of anxiety on vaccination hesitancy could be due to the different psychometric tools used for measuring anxiety. In the present study, GAD-7 was used to assess anxiety, while Bendau and colleagues^19^ used the COVID-19-Anxiety Questionnaire, which measures phobic anxiety symptoms related to the COVID-19 pandemic^35^, and Nazli and colleagues^21^ used COVID-19 Phobia Scale^36^ for measuring fear, anxiety and related behavioural changes during the COVID-19 pandemic. However, Kassim and colleagues^17^ used The Depression Anxiety Stress Scale (DASS^37^), and Alici and Copur^18^ used the Beck Anxiety Inventory (BAI^38^)—both scales were found to yield similar results as GAD-7^39,40^. Therefore, a conclusion as to why anxiety appears not to have played a strong role in the Slovenian postsecondary students’ in their intentions regarding the COVID-19 vaccination cannot be easily made.

An argument could be made that medical students have more vaccine-related knowledge and can therefore make more educated decisions compared to most of the other student groups. This could potentially explain why the fear of COVID-19 and anxiety played a role in the medical students’ intentions to obtain vaccination as soon as it becomes available to them in the future. However, despite healthcare students also having more knowledge relating to vaccinations compared to non-healthcare students, the results in the present study showed high levels of vaccination hesitancy among them. In their review, Mustapha and colleagues^15^ identified various reasons for the healthcare students’ hesitancy, which among others involved the following: vaccination safety concerns (effectiveness, side effects, the rapidness of the vaccine development, etc.), mistrust in the government and their agencies, anti-vaccination beliefs/attitudes. Likewise, Riad and colleagues^16^ identified insufficient knowledge regarding the COVID-19 vaccine safety and government and pharmaceutical industry mistrust as having a significant impact on vaccination hesitancy. Moreover, they found that the availability of vaccines in their local health centre promoted students’ acceptance of COVID-19 vaccination and that the gradual increase in the levels of vaccine acceptance in the USA might be positively associated with the actual availability of vaccination which is proven to be effective and safe^16^.

Considering all of the aforementioned vaccination deterring factors identified by researchers, there are several possible explanations why anxiety did not play a greater role in Slovenian postsecondary students’ expressed intentions to get vaccinated or not. The first potential explanation could be derived from the theory of planned behaviour^30^. The theory proposes that behaviour results from behavioural intentions that stem from attitudes towards the behaviour (i.e. beliefs about the outcome having positive or negative consequences), subjective norms (i.e. perceived social pressure to engage or not) and perceived behavioural control (i.e. one’s ability to carry out the targeted behaviour). The polarisation of the Slovenian public regarding the question of vaccination for COVID-19 (subjective norms), together with postsecondary students being considered as a low-risk group for COVID-19-related complications (attitudes) could have had a greater deterring effect to get vaccinated than anxiety promoting effect to do so. Fear of COVID-19, on the other hand, could have increased the participants’ belief that the cost of not getting vaccinated would be greater than getting vaccinated (attitudes) and thus reduce their vaccination hesitancy.

Secondly, at the time of the present study, the vaccination was not yet available in the local healthcare centres; research on side effects, vaccine effectiveness and potential different outcomes across various groups was still scarce. More specifically, affective forecasting bias, e.g. expectations bias, could have led them to believe that the negative consequences of vaccination for their well-being would be greater than what they really are and thus decide against vaccination. Indeed, the results of the present study showed that anxiety was a positive predictor for the tertiary student group reporting no intentions to get vaccinated in the future, while having no significant impact on the intentions to get vaccinated. Moreover, fear of COVID-19 was found to be a significant negative predictor for having no intentions to get vaccinated and was a significant positive predictor for intending to be vaccinated as soon as possible. Therefore, it appears that some of the students’ anxiety could reflect the mistrust of vaccination as, at the time of the present research, it was not yet available to students at the local health centres^16^ and thus, had a deterring effect on students’ intentions to get vaccinated in the future, especially if the fear of COVID-19 was low at the same time.

Lastly, the anxiety symptoms reported by the participants could be the result of other factors not related to the COVID-19 vaccination. For example, history of anxiety and/or depressive disorder^41^, governmental measurements aimed at reducing the spread of COVID-19, change in studying format (i.e. forced distant online learning)^42^, worries relating to the health of their family members^43^ and worry about social support^44^ have been linked to increased levels of anxiety during the COVID-19 pandemic among postsecondary students.

However, this study had limitations as well. Firstly, it did not explore the influence of various factors on vaccination hesitancy. For example, gender, year of study, urban vs rural living environment, level of COVID-19-related knowledge, financial status and history of mental health difficulties^13,45–48^. All of these factors would offer a better insight into the vaccination hesitancy among Slovenian postsecondary students. Moreover, a psychometric tool designed to measure anxiety particularly related to COVID-19 (e.g., the COVID-19-Anxiety Questionnaire) would enable a better distinction between postsecondary students whose anxiety stems from COVID-19 factors and those whose anxiety emerged as a result of other factors. As this study had a cross-sectional design, a follow-up on the realisation of the expressed intentions by the participants regarding the vaccination was not possible.

Future research should explore more in-depth the influence of various factors on vaccination hesitancy. Furthermore, additional research is needed to determine the effects of the vaccine and COVID-19 knowledge on the vaccination hesitancy as the results of different research are inconsistent^13,46^. Addressing this question would also provide a better insight into why healthcare students express high levels of vaccination hesitancy and medical students do not, while both are considered to have a great amount of knowledge regarding the aforementioned topics. Additionally, as the social norms affect our behaviour, participants’ perception of social norms relating to vaccination hesitancy should be assessed as well when exploring the topic of vaccination hesitancy.

Not only did the COVID-19 pandemic bring numerous changes into our everyday life, but also opened a social dilemma—to get vaccinated or not to get vaccinated for COVID-19. The Slovenian postsecondary students overall reported low intentions to get vaccinated when the vaccine would be available to them and only fear of COVID-19 appeared to have had a small to mild influence on their decision. Even more worrying is the fact that high vaccination hesitancy was observed among prospective healthcare workers as they represent the future gatekeepers and/or promotors of maladaptive behaviours by influencing the health-related decisions of many people. As the numbers are well below the percentages recommended by authorities to allow community (herd) immunity, the decision makers should support further explorations to determine the exact causes for high vaccination hesitancy among Slovenian postsecondary students and subsequent development of targeted interventions and campaigns against it. Moreover, it should specifically explore the reasons behind significantly different levels of vaccination hesitancy among medical students and healthcare students, as the present results might call for a review of the healthcare study programmes in order to ensure a better understanding of the vaccines among future healthcare workers. Not only would these interventions reduce the negative consequences of the potential future outbreaks of a health crisis like COVID-19, but also ensure that the postsecondary students will be the first in line to promote positive health behaviours.

## Data Availability

The datasets generated during and/or analysed during the current study are available from the corresponding author on reasonable request.
